# A prospective cost–benefit analysis for nylon 4N6FLOQSwabs®: example of the process and potential benefits

**DOI:** 10.1007/s00414-022-02884-0

**Published:** 2022-09-03

**Authors:** Bruce Budowle, Jianye Ge, Antti Sajantila

**Affiliations:** 1 Department of Forensic Medicine, University of Helsinki, Helsinki, Finland; 2 Center for Human Identification, Health Science Center, University of North Texas, Fort Worth, TX USA; 3 Department of Microbiology, Immunology, and Genetics, University of North Texas Health Science Center, Fort Worth, TX USA; 4 Forensic Medicine Unit, Finnish Institute for Health and Welfare, Helsinki, Finland

**Keywords:** Forensic genomics, Cost–benefit analysis, Nylon 4N6FLOQSwabs®, Cotton swab, Sexual assault, Rape, Database, Tangible and intangible benefits

## Abstract

Laboratories and their criminal justice systems are confronted with challenges for implementing new technologies, practices, and policies even when there appears to be demonstrative benefits to operational performance. Impacting decisions are the often higher costs associated with, for example, new technologies, limited current budgets, and making hard decisions on what to sacrifice to take on the seemingly better approach. A prospective cost–benefit analysis (CBA) could help an agency better formulate its strategies and plans and more importantly delineate how a relatively small increase to take on, for example, a new technology can have large impact on the system (e.g., the agency, other agencies, victims and families, and taxpayers). To demonstrate the process and potential value a CBA was performed on the use of an alternate and more expensive swab with reported better DNA yield and being certified human DNA free (i.e., nylon 4N6FLOQSwabs®), versus the traditional less costly swab (i.e., cotton swab). Assumptions are described, potential underestimates and overestimates noted, different values applied (for low and modest to high), and potential benefits (monetary and qualitative) presented. The overall outcome is that the cost of using the more expensive technology pales compared with the potential tangible and intangible benefits. This approach could be a guide for laboratories (and associated criminal justice systems) worldwide to support increased funding, although the costs and benefits may vary locally and for different technologies, practices, and policies. With well-developed CBAs, goals of providing the best services to support the criminal justice system and society can be attained.

## Introduction

Forensic genetics, or more appropriately today forensic genomics, continues to be exposed to technological advancements that can improve obtaining DNA profiles from crime scene evidence to support and increase investigative leads and, thus, the overall criminal justice system. Advancements, such as better designed swabs, trace collection procedures, automated extraction instrumentation, enhanced STR kits, Rapid DNA instrumentation, next-generation sequencing instruments and associated chemistries, single-cell analyses, and probabilistic genotyping software, to name a few, offer many advantages but they do come with a cost. In addition to the cost of the new item, there are time and labor resources within the laboratory that must be taken into account for evaluation, validation, and implementation. That overall cost which the laboratory must incur can be a deterrent for consideration of any new technology [see 1 discussion]. One can appreciate this concern and obstacle as laboratories have fixed budgets and dedicating resources to a new capability must compete with limited existing resources. Laboratories often are faced with carving out of existing operational budgets and resources to implement technological advances, which is a difficult decision process with substantial consequences. Alternatively, operating costs must increase.

This approach of viewing the cost of implementing “better” technologies based on a laboratory’s existing budget and what it can absorb may not be the ideal way to proceed. Better is defined herein as enhancing the ability to obtain a genetic profile that supports an investigative lead(s), and that laboratory is used throughout but depending on the technology or policy a greater part of the criminal justice system may need to absorb the cost. Instead of an inward-looking strategy, it can be more effective and more convincing to those who approve overall budgets for the laboratory if a cost–benefit analysis (CBA) with a prospective systems approach is performed and the benefits are described. There have been some forays into performing a CBA in forensic science on the value, for example, of analyzing all sexual assault kits, but mostly after the policy was enacted [2–8]. Implementation and operation of national databases, especially early adopters, likely were supported qualitatively with the belief that they would assist in solving crime, exonerating the innocent, and most importantly reducing future crime as opposed to performing a formal CBA to justify the expense. Indeed, hundreds of millions of dollars have been invested into national database systems because of the perceived tangible and intangible benefits they bring (although without a CBA). Retrospective analyses, however, tend to support that the investment in databases was justified [2–7].

A CBA, used in economics and increasingly in healthcare [for example, see 9, is an assessment of the costs associated with a particular technology, process, or policy compared with the benefits (i.e., cost savings) that an individual, agency, or society may gain. Usually, these costs and benefits are couched in monetary value, but at times they can be assessed qualitatively, such as was likely done for database implementation, when such data were not accessible. The primary reason for performing a CBA is that with limited resources a laboratory (or criminal justice agency) needs to justify that the investment is effective in providing the greatest good to the most people who may be impacted. Performing such an exercise also allows the laboratory to plan and execute a process in a more effective manner. The benefits are translated into costs as monetary value is understood and readily compared by those who have to make financial allocation decisions.

According to Henrichson and Rinaldi [10] there are six steps in performing a CBA (Table 1). These steps are similar to those of other CBAs [11, 12]. They can be performed sequentially, and some steps may be combined.

**Table 1:** Steps and general considerations associated with performing a cost–benefit analysis^#^

Steps	Process	Considerations
1	Identify and assess the potential impacts of the investment	Is the program feasible? Does it accomplish the desired goal within the lab? Does it impact other sectors? Are there similar initiatives that have been successful?
2	Measure the costs of the investment	What are the costs to carry out the program within the laboratory?
3	Measure the costs and benefits of the investment’s impacts	What is the monetary value of the investment’s impacts? Who benefits from the program? Who bears the costs within the laboratory and other impacted sectors? What is the magnitude of the effect for each relevant sector?
4	Compare costs and benefits	Do the benefits justify the costs (short and long term)? Does the investment in the laboratory operation deliver higher or lower returns than the current system or alternate approaches (if available)?
5	Test the reliability of the results	Are the assumptions made to predict the expected costs and benefits justified? If assumptions are varied what is the impact? Would different information change the predictions substantially, modestly, or not at all?
6	Report the results	What are the ways to tabulate the findings and manner to best present the findings and conclusions?

^#^Process and considerations derived and modified from Henrichson and Rinaldi [10]

It can be readily argued that the cost of the laboratory process is trivial in the overall benefits to the criminal justice system and to crime victims (including family and community) both in tangible and intangible ways [2–8]. Laboratories should consider performing CBAs to show in a monetized manner (or qualitatively if data are not available) of how the relatively small increase of their budgets can have large impact on the criminal justice system and society. In addition, a CBA would be extremely invaluable for large investments, such as enhancing databases or converting to massively parallel sequencing technologies.

To demonstrate the logic, the process, and some of the considerations, we performed a CBA on the use of an alternate and more expensive swab with reported better DNA yield and being certified human DNA free (i.e., nylon 4N6FLOQSwabs®) versus the traditional less costly swab (i.e., cotton swab). It is important to point out the strengths and limitations of the assumptions that support a CBA and whether there is support, if available, from other studies. The same exercise could be performed on any potential better technology, but swabs were selected because of the simple concept and the assumptions were easy to entertain. The overall outcome is that cost of using the more costly technology pales compared with the potential benefits, tangible and especially intangible ones.

## Materials and 0ethods

Data used to support the performance and costs are cited in the paper. Collectively these resources provide data on enhanced performance of the swab, database (e.g., CODIS) performance, casework load, and costs for operations and costs to society. Costs of swabs were obtained from vendors. Costs and benefits were based on assumptions (low and high) to derive monetary value. Descriptive statistics were used to derive the cost benefits.

## Results and discussion

The potential impact proposed for this CBA is that a swab that yields more DNA from a crime scene sample will (1) contribute to more DNA profiles uploaded to a DNA database, which in turn (2) increases offender and forensic hits, (3) increases the investigative leads supported to solve crimes, and (4) because of recidivism reduces future crime that will have notable cost savings and intangible benefits to the greater society (first and foremost to victims and families as well as to the criminal justice system and to taxpayers). It is unknown at this time if such increased efficiency would improve database searches with mixture evidence that does not have a resolvable major or minor component as it clearly would with resolvable major profile evidence. However, recent work with probabilistic genotyping [13] would suggest that an improvement could be attained with more complex mixtures as well.

The 4N6FLOQSwabs® (COPAN, Brescia, Italy) is a nylon-flocked swab (certified human DNA free) that, in theory, because of its design, should outperform cotton and rayon swabs on collection and yield of crime scene DNA. Early studies by Benschop et al. [14] (a forensic study), more recent studies by Viviano et al. [15] (a non-forensic study), and others [16–20] support the proposition that the nylon swab collects and yields more DNA, although some studies have come to different conclusions [21, 22]. Differences in performance between studies appear to be due to varying protocols that may not be the one validated by the manufacturer for the nylon swab, or for that matter the compared swabs as well [1, 16]. Nonetheless, the better performance information of the nylon swab is illustrative for performing this CBA. Viviano et al. [15] testing for HPV observed improved performance in target detection with the flocked swab versus a cotton swab but importantly also obtained an ~ 4.4-fold increase in recovery of vaginal epithelial cells. This finding supports that the nylon swab provides a substantial increase in DNA yield. While this study measured vaginal cell recovery, the study by Benshop et al. [14] indicates that more male DNA can be recovered with the nylon swab. The increase in yield may not be linear, for example, for trace or touch DNA but is indicative of an increased yield (especially coupled with other studies such as Benschop et al. [14]). Thus, it would seem reasonable to assume that replacing a cotton (or rayon) swab with the nylon swab, and following optimized protocols, would improve DNA typing success and result in more profiles uploaded to a DNA database. There are no data (of which we are aware) that allow one to estimate accurately how many more profiles may be uploaded due to increased recovery of DNA. Data such as the number of samples that yielded DNA that was just insufficient to yield the minimum number of short tandem repeat (STR) markers necessary to meet upload requirements would be extremely beneficial. But reasonable assumptions and a range of low and high expectations can be made.

To replace the current swabs there is a cost related which is primarily that of purchasing the swab. We do not consider herein training costs in this analysis as current swab users should be proficient in swabbing, newly hired people would be trained regardless of the type of swab, and the differences in usage of swabs (that do exist) would be nominal. Nor do we consider herein the cost to validate (or more appropriately verify) the use of the nylon swab which should be small (but one can add in the verification costs to replace currently used swabs if desired). The costs for a nylon, cotton, and rayon swab are $0.66 for the version in a pouch up to $1.23 for the version in a tube, $0.15, and $0.09 per swab, respectively (prices obtained from vendors). These values are approximate and do not necessarily reflect the cost if purchased in bulk or via vendors. Nonetheless, the point here is that use of the nylon swab (in a tube) will cost ~ 8 to ~ 14 times that of a cotton or rayon swab, respectively.

One then can consider the impact of a wholesale replacement of crime scene collection swabs with the more expensive nylon swab. This impact likely will be borne by a number of groups including law enforcement, sexual assault nurse examination programs, the laboratory, and others. The impact of a wholesale swab replacement would depend on the number of cases and number of samples collected using swabs. The Bureau of Justice Statistics (the most recent data from 2014) [23] provided statistics for the USA on casework requests for analysis of biological evidence. In that year, there were 333,000 casework requests of which 45,000 were for sexual assault analyses. These numbers may be reasonable for a CBA, but there is a possibility that not all crime scene evidence is submitted to crime laboratories and thus the number of cases and hence the number of swabs used could be an underestimate (not factored into this CBA). It is worth noting that less swabs might be taken if, for example, a higher yield swab was employed, which in turn could reduce the swab cost and subsequent laboratory operation cost, which is not factored herein. Based on our experience, 5 swabs per case were considered (which is consistent with estimates reported between 4 and 7 [6, 24]); the cost for swabs (using the values stated above for each swab) for the 333,000 cases (and the subset of 45,000 sexual assault cases) is shown in Table 2. The cost difference nationwide for wholesale replacement of cotton with the nylon swab in a tube (i.e., the higher price range of the 4N6FLOQSwabs®) would be $1,798,200 (rayon is not the primary swab currently used in the USA). For sexual assault cases, the cost difference for the swabs would be $243,000. Of course, these differences would be less for individual local, state, province, or federal systems, as this cost would be borne across the jurisdictions.

**Table 2:** Costs for swabs associated with casework requests^#^

Swab type	Cost for all casework requests^*^	Cost for all sexual assault casework requests^**^
Rayon	$149,850	$20,250
Cotton	$249,750	$33,750
Nylon^***^	$2,047,950	$276,750

^#^Casework request numbers derived from Bureau of Justice Statics [23]

^*^Costs are based on 5 swabs for each of 333,000 cases, assumes that only one swab type is used by all evidence collectors.

^**^Costs are based on 5 swabs for each of 45,000 cases (which are a subset of the 333,000 cases), assumes that only one swab type is used by all evidence collectors.

^***^The nylon swab in a tube price of $1.23 per swab was used for calculations.

The next part of this CBA is to consider the number of investigative leads (i.e., in this scenario is the number of hits) developed through a database search. Other cases in which a suspect profile is compared one-to-one with the evidence profile may not be captured herein, but since laboratories routinely search such cases against the database for potential hits to other unresolved cases, the effect may be small. Using statistics from CODIS [25], as of October 2021, there were 1,144,255 forensic profiles which have returned 587,773 hits over the life of the database, which translates to ~ 51% hits. This number is not based on one hit to one sample as multiple hits to a sample may be counted only once but is used for illustrative purposes and would not be substantially different. Although CODIS has been in operation for almost 24 years, the number of forensic profiles uploaded per year was derived using 10 years as the denominator as more samples have been uploaded disproportionally in more recent years and likely would represent more current (increased) upload numbers. Thus, for this exercise 114,426 forensic profiles were estimated to be uploaded per year. With 333,000 cases requested per year and assuming 114,426 profiles uploaded per year and one profile per case (which may not be an accurate assumption), the upload to request ratio is 0.34. This number is not dramatically different than the uploads reported for the National Sexual Assault Kit Initiatives (SAKI) [26]. From 2015 to present SAKI has enabled 72,350 sexual assault kits to be completed and these kits have resulted in 30,259 DNA profiles uploaded to CODIS. Thus, the SAKI upload to completed kit ratio is 0.42.

This rate and number of uploaded profiles are conditioned on that the samples yielded sufficient DNA to generate a minimum of markers per profile to be entered in a database. Data are not available to ascertain what portion of 66% (based on 0.34 upload proportion) or 58% (based on 0.42 upload proportion) of kits (assuming one kit per case) that were not uploaded were due to complexity (i.e., higher order mixtures) or were marginally deficient in recovered DNA and would have yielded an acceptable profile to upload if DNA yield was increased using the higher performing swab. But given the demonstrated increased DNA recovery with the nylon swab one could assume, while arbitrary, a low of 10% increase and a high of 30% increase. There are little data to refine the potential increase. For example, information regarding DNA yield on samples that did not generate an uploadable DNA profile would be instrumental in predicting the percent increase. Laboratories should collect quantitative data to facilitate estimating potential success with various technology enhancements. Regardless, alternate percent increases can be entertained if desired to determine the CBA. The rest of the CBA focuses on sexual assault cases because there are other CBA data available for comparison and support of our assumptions [2–8, 27]. Other crime categories can be added following similar approaches and applicable assumptions.

Assuming a similar proportion for sexual assault kit DNA typing success to the upload proportion per case calculated above, i.e., 0.34 which is lower than the SAKI data, the number of additional profiles uploaded nationwide based on a 10–30% increase is shown in Table 3.

**Table 3:** Number of additional profiles uploaded from sexual assault cases due to increased DNA yield

% increase of profile uploads	Number increase of profile uploads^*^
10	1530
20	3060
30	4590

^*^Calculated as 45,000 cases × 10, 20, or 30% increase × 0.34.

With the data in Table 3, the costs associated with the increase in uploads of sexual assault profiles can be estimated. The tangible and intangible costs reported by Miller et al. [28] associated with crime are used as they represent national numbers and address many crime categories. These overall costs compare similarly with other studies [for example, see 27,29]. Miller et al. [28] estimate the tangible costs (i.e., medical, mental health, productivity, property loss, public services, adjudication and sanctioning, and perpetrator work loss) related to a rape at $11,923 per incident and intangible (i.e., quality of life) costs at $214,518 per incident. Note that intangible costs are substantially higher than tangible costs and have the greatest impact on victims and society (Table 4). It also is worth noting that society values the intangible benefits related to the safety and security of its victims [for example, see 30]. We focus on three of the tangible costs — medical, public service, and adjudication and sanctioning — because these are the ones most readily identified as cost impact that could be increased with more hits and could save resources with more hits. The tangible costs for these three categories per rape are $1835, $25, and $852, respectively. The $25 for public services (includes investigation costs) seems low [for example, see 7] but is used anyway. The costs associated with these cases are shown in Table 4. Medical treatment would occur regardless of DNA typing and thus it does not change for the victims (at this point of the analysis). The monetary value in Table 4 for public service and adjudication and sanctioning costs assumes that all uploads result in a hit and are further investigated, adjudicated, or dismissed. Data from the SAKI for sexual assault cases indicate that the hit proportion to profile upload is 0.46 (13,961 CODIS hits/30,259 uploaded profiles). There were 20,448 investigations associated with SAKI likely indicating that CODIS hit and no-hit cases are investigated, but only 1874 cases were charged. Those increased uploaded profiles that garner a hit would impact the workload of other parts of the criminal justice system which is a cost that must be borne by other portions of the criminal justice system. However, as noted by Lovell et al. [27] the criminal justice system is committed to pursuing all cases with lead value (again in part because of the value of intangible benefits to society) and thus an increase in such work need not necessarily be factored into safety and security herein (if desired, the cost, which was not done in this study, could be readily calculated using the data in Miller et al. [28]). Indeed, such factors can be taken into account if one desires as it could be considered additional costs that must be borne with more database hits. Interestingly, Lovell et al. [27] presented that database hits increased the time investigators dedicated to the investigation (~ 40 h/case) compared with cases that did not yield a hit (~ 20 h/case). Davis et al. [7] estimated the hours/case to be similar at 32.64 and 12.72, respectively. A premise for use of DNA databases was that a hit would reduce the time and resources for investigators to develop a good lead. In contrast, the opposite may be occurring. Perhaps with hit information, investigators have good leads and thus motivation to put more effort into a case. We are not sure if more or less effort is generated with a database hit but agree with Lovell et al. [27] that the criminal justice system would absorb the work for the safety and security of society and thus do not factor that cost in this CBA.

**Table 4:** Tangible costs^#^ associated with sexual assault cases and increased profiles uploaded to a database

Number of sexual assault profiles uploaded	Medical cost ($1835)	Public service^##^ cost ($25)	Adjudication and sanctioning cost^##^ ($852)	Total cost^##^
1530	$2,807,550	$38,250	$1,303,560	$4,149,360
3060	$5,616,100	$76,500	$2,607,120	$8,299,720
4590	$8,422,650	$114,750	$3,910,680	$12,448,080

^#^Cost per category derived from Miller et al. [28]

^##^Assumes that all cases are subject to these categories, which is unlikely. However, the data in Miller et al. [28] factored the monetary value overall cases.

The real cost benefit in a tangible way is the reduction of future crime, which in this CBA are sexual assaults by serial offenders. The exact number of stranger assaults that are serial offenders is unknown, but some studies have estimated it to be from about 8 to 25% of hits [see discussion in 27]. Data from the SAKI indicate that 1938 CODIS hits out of the of 13,961 hits were to serial sex offenders which is 13.9% of all hits related to the initiative. This percentage of hits is likely underestimated (see below). These hits, if acted upon early on because of increased DNA typing success, would reduce the number of victims and thus save in, for example, medical costs. Database success is based on recidivism and solving future crime through DNA typing. Based on SAKI (operational) recidivism through a database search could result in 5 to 15% (low or modest) hits associated with repeat sexual assault offenders. If these cases were prevented, the tangible and intangible savings would be quite notable (Table 5) ranging from $208,824 to $1,865,856 for this sub-portion of tangible costs and a total cost benefit ranging from of $16,726,710 to $149,454,240. This amount of savings is comparable to other studies. For example, Wang et al. [6] suggested that for every dollar spent on sexual assault kit analyses results in a saving of $81.34 associated with future sexual assaults that are prevented. Wickenheiser [8], using more optimistic assumptions, suggested a cost savings between $63.79 and $249.68 per dollar spent. Although the assumptions and process to derive their cost benefits are different and may not be directly applicable, this cost benefit value was considered. With an increased expense of $1,798,200 for the use of nylon swabs the cost benefit using $81.34 would be predicted to be $145,723,864 which is comparable to the values in Table 5.

**Table 5:** Costs^#^ associated with serial rapes that may be captured with increase upload of profiles to a database

Benefits	77 victims^##^	688 victims^###^
Medical	$141,295	$1,262,480
Public service	$1925	$17,200
Adjudication	$65,604	$596,176
Total tangible^***^	$208,824	$1,865,856
Intangible	$16,517,886	$147,588,384
Total tangible and intangible	$16,726,710	$149,454,240

^#^Cost per category derived from Miller et al. [28]. Note that a sub-amount of total tangible costs only considering those categories listed in this table.

^##^Calculated as 0.05 × low end estimate (10%) of increased uploads. Note that 77 victims are 5% of the 1530 uploaded profiles in Table 3

^###^Calculated as 0.15 × high end estimate (30%) of increased uploads. Note that 688 victims are 15% of 4590 profiles uploaded from Table 3

^***^Total tangible is the sum of medical, public service, and adjudication costs.

For the cost benefits in Table 5 to be realized most hits need to be acted upon. However, the data for sexual assault cases would suggest otherwise. Data from SAKI indicate that of the 72,350 kits in which testing was completed there were only 20,448 investigations. So, for this CBA, a correction on the cost benefits might be the portion that generate investigations. Assuming that 28% (20,448/72,530) were acted upon the cost benefit could be reduced proportionally to $58,471 to $522,440 for this sub-portion of tangible costs and a total cost benefit ranging from $4,683,479 to $41,847,187 (0.28 × $16,726,710 or $149,454,240, respectively). These values are likely low as they do not capture the full value of uploading a DNA profile which include but are not limited to future hits, linking cases with no suspect, exonerations, investigative information from cases beyond the statute of limitations, and commitment by government to address crime and particularly violent crime.

The increase of costs to the laboratory operation subsequent to DNA recovery from the swab were not factored into the CBA as likely they would be small because most case requests are worked, and typically laboratories proceed with typing even when the quantity is low and/or with a high degradation index. It is conceivable that there could be an increase in some DNA typing, i.e., amplification and STR typing. One could add the cost by determining the cost of STR/PCR reactions and labor involved if it is deemed that the cost would be substantial. However, that cost could be offset by less sample manipulation (such as analyzing fewer swabs to obtain sufficient DNA from multiple swabbings or by reducing the need to re-sample an item of evidence if the first sampling did not yield sufficient DNA for downstream analyses) due to increased DNA yield by using a better performing swab.

As with any such analyses there are considerations and limitations to report with this CBA. They are.

- This CBA was not meant to be comprehensive and capture all costs as well as some assumptions may not be accurate. The assumptions were based on extant data and personal experience, but data are limited. The intention, however, was to show the process and its complexity (or for that matter its simplicity), but that such analyses can provide reasonable support for increasing a laboratory’s budget for the betterment of the criminal justice system and society. If more quantifiable data were available, the costs and cost benefits could be better estimated, although with this swab study the overall outcome of cost benefit would still be supported. Other technologies, such as massively parallel sequencing and Rapid DNA technologies, would require other data as well. We urge laboratories and their criminal justice systems to collect more data from their operation performance (casework and database) as it would help them in performing CBAs.
- The proposition that the 4N6FLOQSwabs® swab outperforms a cotton swab was based on assessment of the literature, how analyses were performed in those studies, and personal experience. The most likely explanation for conflicting results is that optimized protocols were not used per swab type. It is recommended that laboratories (initially) should perform a small verification study under recommended previous validated protocols to determine swab effectiveness.
- The cost differential between that of the 4N6FLOQSwabs® and that of the cotton swab may be less than what was reported herein. The price of the swab in a tube was used for this CBA as it was likely a high end value.
- The total tangible costs from Miller et al. [28] were not used in this CBA; only a subset of tangible categories was considered for simplicity and obvious impact on services. The categories were medical, public service, and adjudication and sanctioning. The total tangible cost for these three categories is $2712. The total tangible cost associated with rape was reported at $11,923. The tangible cost used in this CBA is 4.4-fold less. Thus, the potential numbers for this aspect are likely low estimates.
- Cases not uploaded may have “relatively” low quantity DNA evidence and increased yield should have a great impact on generating profiles uploaded to national databases. The upload proportion value for sexual assault casework was estimated at 0.34. This value is lower than that observed from SAKI. It can be expected that the upload value is higher with usage of a better performing swab and also would increase with a test all initiative.
- The process herein was based on limited USA data and may or may not be generalizable within the USA or for other countries. However, the CBA process for each laboratory still would be similar and the process described herein could help guide laboratories worldwide. Most importantly, the tangible and intangible benefits gained can be quite notable and substantial compared to the outlay of laboratory operational costs. Thus, some latitude in assumptions can be tolerated, and the findings in this CBA may be generally applicable.
- Sexual assault cases were the focus of this CBA as there were some accessible supporting data. These costs to society for sexual assault analyses are likely low estimates because the number of rape cases reported is low (25% and possibly as low as 5% or less) [2, 28, 31] and yet medical care, for example, may still be pursued. Those victims that do not report also could be impacted, assuming that a percentage of such cases were perpetrated by a serial rapist. Also, since the casework statistics were generated, there have been major efforts in various states to address the sexual assault kit backlog and have instituted a test all policy. Therefore, the number of uploads and thus hits used herein may be an underestimate.
- Increased success with DNA typing and more hits being investigated could provide more confidence to victims and which in turn could increase the reporting rate. This increase is an example of a qualitative benefit that could have been monetized but the assumptions would be merely educated guesses.
- More cases uploaded, which in turn yields more hits, provide resolution to victims and safety and security to victims, families, and communities. This intangible benefit related to quality of life is one of the primary motivations of the criminal justice system, the government, and the people and should be factored into CBAs, at least qualitatively.
- Increased DNA yield facilitates the laboratory process for sample screening and can reduce sample manipulations (e.g., re-extraction, DNA concentration, re-typing) (i.e., can streamline) to achieve sufficient DNA analysis results which could be factored into a benefit on laboratory operations.
- The analyses are based on uploads to CODIS which may not capture all cases analyzed and thus likely results are an underestimate of benefit costs.
- This CBA focused on sexual assault cases (using tangible and intangible costs associated with rape). To be more comprehensive on the potential impact similar analyses can be performed for murder, assault, other sexual assault, burglary, etc. Since these case types are the majority of the 333,000 cases worked per year and are being worked, the overall benefits are much larger than those estimated herein solely for sexual assault casework.
- Differences to consider in a CBA for other crimes may be, for example, the overall tangible and intangible costs per murder were higher than per rape, but there were fewer murders, and there were twice as many assaults reported than rapes and costs were similar (~ > 0.75) but not all may leave biological evidence.
- One aspect that was not factored is the benefit of uploading samples (even if they do not retrieve a hit initially). Populating the database does have a benefit of increasing the hit rate.
- Another aspect not factored is the value of exoneration. In some cases of wrongful convictions and wrongful arrests searching the database has provided leads to other more likely suspects [see 32]. It is conceivable that the tangible and intangible costs of wrongful convictions and wrongful arrests on a per case basis rival if not exceed those of rape and murder, especially when considering the social stigma, loss of freedom, loss of productivity, pain and suffering, and subsequent settlements.
- There are other values that were not monetized that can have benefits to the system. For example, the 4N6FLOQSwabs® is certified human DNA free. This quality of the swab reduces contamination and thus could reduce compromising samples, reduce false lead efforts, and limit litigation [see 33,34] especially in an adversarial system. Another benefit in design is that the handle has a pre-marked breaking point to facilitate placing the swab head into a tube for extraction.
- There are other costs and benefits to consider, such as opportunity costs and competitive benefits [12]. There can be lost opportunities when one approach is taken over another or by maintaining the status quo. The laboratory may not be seen as, for example, a center of excellence or a leader by those who allocate funds if better technologies are not implemented and other laboratories are doing so. Being a leader could gain a competitive edge for grant funds that support current operations and new endeavors. Additionally, customers may be willing to pay a greater price for a service if the laboratory is considered a center of excellence.

In conclusion, this study shows that the $1,798,200 increase in 4N6FLOQSwabs® swab cost per year pales in comparison with the cost benefits (monetized and qualitatively) by increasing the number of uploaded profiles, obtaining more hits, and preventing future victims. Thus, “going cheap” in one part of the system is not necessarily better for the whole system. With this swab CBA there were assumptions made and one can debate whether any or all of the assumptions are valid. However, as noted throughout some potentially were underestimated, others overestimated, and some were qualitatively considered with the intent of being demonstrative and showing what may need to be considered. If desired, a different value can be inserted, and that cost effect can be determined using the process. Some aspects were not included as they may be better to assert qualitatively in addition to the quantitative values. A similar CBA approach could be performed and potential benefits on a system level could be realized for any potential technology, for example, undertaking cold case initiatives or building an alternate database system, with similar and different tangible and intangible categories and costs. This approach could apply to laboratories (and associated criminal justice systems) worldwide to support increased funding. It would be beneficial for laboratories from a strategic planning perspective and from a justification perspective for increasing their budgets to perform a CBA(s). To facilitate a CBA, it also would be beneficial for laboratories and criminal justice systems to capture performance data (for casework operations and database operations) relevant to necessary assumptions. With such data effective CBAs can be generated, and the laboratory can achieve its goals of providing the best services to support the criminal justice system and society.
